# Statins and Thyroid Eye Disease: A Propensity Score-Matched Retrospective Cohort Analysis

**DOI:** 10.7759/cureus.83243

**Published:** 2025-04-30

**Authors:** Maxim J Barnett, Carlo Casipit, Justin Lam, Ana Rivadeneira

**Affiliations:** 1 Internal Medicine, Jefferson Einstein Hospital, Philadelphia, USA; 2 Endocrinology and Diabetes, Jefferson Einstein Hospital, Philadelphia, USA

**Keywords:** exophthalmos, graves' disease, graves' orbitopathy, statin use, thyroid eye disease (ted)

## Abstract

Background

Thyroid eye disease is a sight-threatening disorder associated with Graves’s disease. While treatment is aimed at stabilizing established thyroid eye disease, there are limited investigations regarding prevention. Statins, known for their anti-inflammatory properties, are one such treatment that has been investigated for thyroid eye disease. This study retrospectively analyzes the impact of statin medications on thyroid eye disease and related outcomes from real-world data using TriNetX Global Collaborative Network (TriNetX, Cambridge, MA, USA).

Objective

The primary objective of this study is to analyze the risk of developing thyroid eye disease in patients with Graves’ disease treated with statin medications relative to those without exposure up to five years. Secondary objectives of this study include analyzing thyroid eye disease-related outcomes, including diplopia, optic nerve surgery, optic nerve injury, strabismus, blindness, requirement for teprotumumab, color vision deficiencies, and referral to ophthalmology.

Methods

Data were collated utilizing TriNetX Global Collaborative Network, a platform of globally de-identified patient information from over 140 healthcare organizations. Patients more than 18 years of age with a diagnosis of Graves’ disease (ICD-10: E05.0 Thyrotoxicosis with Diffuse Goiter) were divided into Cohorts A (administration of statin medication following diagnosis of Graves’ disease, n = 66,444) and B (no statin medication administration, n = 225,905). Via implementation of propensity score matching to control for confounding factors, a total of n = 43,025 matched patients per cohort was obtained. Hazard ratios (HRs) and 95% confidence intervals (CIs) were used to assess the primary and secondary objectives over a five-year follow-up period. The t-test was used for the calculation of mean averages of ophthalmology referrals and to determine statistical significance.

Results

Patients treated with statin medications (Cohort A) demonstrated a significantly lower HR for the development of exophthalmos (HR = 0.559, 95% CI 0.521-0.601, p < 0.001). Statin medications were furthermore associated with a reduced HR for diplopia (p < 0.001), optic nerve surgery (p = 0.018), and requirement for teprotumumab (p = 0.047). No significant reduction in hazards for color vision deficits, strabismus, optic nerve injury, or blindness was noted. Additionally, there was no significant difference in referrals to ophthalmology.

Conclusion

The findings from this study suggest that statin therapy may be protective against the development of thyroid eye disease and associated complications. Further randomized controlled trials are required to validate these findings and establish a therapeutic role for statins in thyroid eye disease.

## Introduction

Thyroid eye disease is the most frequent extrathyroidal manifestation of Graves’ disease, present in up to 50% of patients [1]. The disease itself is not entirely understood but believed to be due to an underlying autoimmune pathogenesis from shared antigens within the thyroid and orbit, with resultant fibroblast activation and orbital inflammation [1]. While a myriad of signs and symptoms can be experienced, however, the most common manifestation is the finding of upper eyelid retraction (present in more than 90% of patients), followed by exophthalmos (proptosis, present in 60% of patients) [2]. Notable risk factors for thyroid eye disease include race (African American), gender (female preponderance), age (bimodal peak incidences among both genders), smoking, radioactive iodine ablation, thyroid-stimulating immunoglobulin titers, and pregnancy [3]. In 1945, Felix Rundle proposed a curve to predict the trajectory of thyroid eye disease, which is eponymously known as “Rundle’s Curve”; the course is biphasic, with an initial active (proliferative) phase lasting up to 18 months, followed by a decrease in severity before plateauing in the quiescent (stable/fibrotic) phase [4]. Early interventions within the active phase are recommended to reduce the severity of symptoms and decrease the risk of the feared complication of dysthyroid optic neuropathy; whereas, within the fibrotic phase, surgical intervention is the only treatment modality offered. Numerous guidelines provide recommendations for management of thyroid eye disease based on the severity as demonstrated by the European Group on Graves’ Orbitopathy (EUGOGO) classification; current options include selenium supplementation, corticosteroid therapy (prednisone or methylprednisolone), steroid-sparing agents (cyclosporine or azathioprine), rituximab, tocilizumab, or radiotherapy [5]. In January 2020, teprotumumab became the first medication to receive Food and Drug Administration (FDA) approval for thyroid eye disease [6].

At present, however, there are no approved treatment options for the sole prevention of thyroid eye disease. Within the literature, statin medications have been proposed to lead to a reduction in the incidence (and progression) of thyroid eye disease, however, this appears to be limited to seven studies (one randomized controlled trial and six observational studies). We perform an observational study to validate such findings, and to contribute to the scant literature pertaining to this topic.

## Materials and methods

Study design and data source

As noted, limited data are investigating the role of statin therapy in the prevention of thyroid eye disease. Of the currently published studies, most are limited to a geographic region (Italy, Sweden, Korea, Taiwan, and the United States of America). This study is performed without a geographic restriction, encompassing global data to power external validity. Data were acquired through TriNetX Global Collaborative Network (TriNetX, Cambridge, MA, USA), a platform of global de-identified electronic health records encompassing more than 200 million patients across more than 140 healthcare organizations (both inpatient and outpatient settings). Currently, 21 countries are included in the TriNetX network (Australia, Belgium, Brazil, Bulgaria, Estonia, France, Georgia, Germany, Ghana, Israel, Italy, Japan, Lithuania, Malaysia, Poland, Singapore, Spain, Taiwan, United Arab Emirates, United Kingdom and the United States of America). As de-identified patient information was used, an institutional review board was not required for this study.

Diagnoses for our study were identified using the 10th Revision of the International Statistical Classification of Diseases and Related Health Problems (ICD-10) codes (and the ninth revision when no further code was available). Additionally, medications were identified by the United States National Library of Medicine RxNorm codes, and procedures were identified by the ICD-10 procedure coding system (PCS), Current Procedural Terminology (CPT), or Systematized Nomenclature of Medicine (SNOMED).

Study population

The population analyzed in our study was those with a diagnosis of Graves’ disease (ICD-10 Code: Thyrotoxicosis with Diffuse Goiter; E05.0), above the age of 18. All genders were included in this study, without a time restriction, as well as both inpatient and outpatient settings; by default, per the TriNetX platform, without a time-restricted period, patients were included between the years 2006 and 2024. Cohorts were divided based on the initiation of a statin medication following diagnosis of Graves’ disease (Cohort A) and those who did not commence statin medication following a diagnosis of Graves’ Disease (Cohort B). All statins were included in the analysis: Pravastatin (RxNorm 42463), Fluvastatin (RxNorm 41127), Rosuvastatin (RxNorm 301542), Lovastatin (RxNorm 6472), Pitavastatin (RxNorm 861634), Atorvastatin (RxNorm 83367), and Simvastatin (RxNorm 36567). Past users of statin medications were excluded from both groups.

Statistical analysis

Data were collected upon the fulfillment of the criteria set by the cohort, which was indicated as the “index event,” with outcomes counted only thereafter. Baseline data before and after matching were collected. Propensity-scoring was employed to allow for matching for an even balance among Cohorts A and B. Cohorts were balanced for 20 characteristics: demographics (Age at Index, Male, Female, White, African American), diagnoses (Tobacco Use, Cerebral Infarction, Ischemic Heart Diseases, Hyperlipidemia, Chronic Kidney Disease), procedures (Thyroidectomy, Radioactive Iodine Therapy), medications (Propylthiouracil, Methimazole, Methylprednisolone), and laboratory values (LDL-Cholesterol, Thyroid-Stimulating Hormone (TSH), Thyroid-Stimulating Immunoglobulin, HbA1c, Body Mass Index Percentile). To minimize confounding, a 1:1 propensity score matching method was applied. Kaplan-Meier Survival Analysis (with log-rank test), hazard ratios (HRs), 95% confidence intervals, and p-values (considered significant when less than 0.05) were obtained both before and after propensity-score matching. For the calculation of the secondary outcome of ophthalmology referrals, a t-test was used to assess for statistical significance.

Outcomes

The primary outcome of this study was to assess the development of thyroid eye disease (specifically, exophthalmos) in patients with Graves’ disease over five years in those treated with statin medications compared to those without exposure (ICD-10: H05.20, H05.2, ICD-9: 376.21). The secondary outcomes of this study were to assess thyroid eye disease related outcomes including optic nerve surgery (ICD-10 PCS: 00NG4ZZ, 00NG3ZZ, 00NG0ZZ; SNOMED: 171652004; CPT: 6750, 31294), optic nerve injury (ICD-10: H46.9, H47.099, S04.01, S04.019, S04.019A), Color Vision Impairment (ICD-10: H53.5), Diplopia (ICD-10: H53.2), Blindness and low vision (ICD-10: H54), Strabismus (ICD-9 CM: 378), Teprotumumab prescription (RxNorm: 2274803), and Ophthalmology Referrals (SNOMED: 183543004, 183699009).

## Results

The initial search obtained datasets from 138 healthcare organizations for Cohorts A and B. Before matching, our initial search identified n = 282,221 (Cohort A, n = 64,975; Cohort B, n = 217,246). Geographic distribution of Cohort A demonstrated 90% within the United States of America, with the remaining 10% among the 21 countries available in the TriNetX platform (breakdown per country outside of the United States of America not individually listed). Concerning Cohort B, the United States of America accounted for 77%, compared with 23% among the 21 other countries (breakdown per country outside of the United States of America not individually listed).

**Figure 1:**
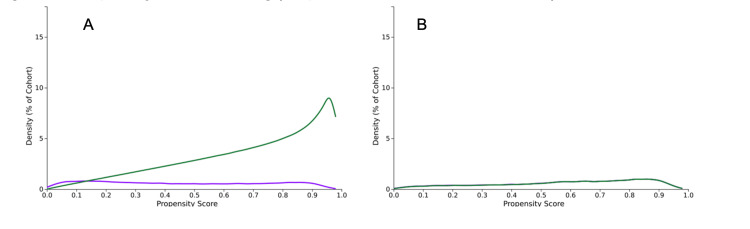
Propensity score matching Graphical representation of the density (percentage of cohorts) before and after propensity score-matching. (A) Before propensity score-matching (purple: Cohort A (treated with statins); green: Cohort B (not treated with statins)). (B) After propensity score-matching (one-to-one matching).

After 1:1 matching, we obtained n = 43,025 per cohort (total n = 86,050), which were well balanced across key demographic and clinical variables (Figures 1A, 1B). After matching, Cohort A demonstrated a mean age of 60.3 ± 12.4 compared to 61.7 ± 13.6 in Cohort B (p < 0.001, Standard Difference 0.106). Further comparisons included African American (Cohort A 17.1% versus Cohort B 16.6%, p = 0.035, Standard Difference 0.014), Female Gender (Cohort A 73.9% versus Cohort B 74.0%, p = 0.762, Standard Difference 0.002), Tobacco Use (Cohort A 4.2% versus Cohort B 3.9%, p = 0.087, Standard Difference 0.012), and Methimazole Treatment (Cohort A 24.8% versus Cohort B 24.3%, p = 0.115, Standard Difference 0.011) (Table 1). Among Cohort A, statin medications were as follows: Atorvastatin (61%, n = 41,826), Rosuvastatin (24%, n = 16,543), Simvastatin (18%, n = 11,927), Pravastatin (12%, n = 8,257), Pitavastatin (2%, n = 1,649), Lovastatin (2%, n = 1,410), and Fluvastatin (< 1%, n = 312).

**Table 1:** Characteristics of each cohort before and after matching HbA1c: Glycated Hemoglobin; IU: International Units; LDL: Low-Density-Lipoprotein; mg: Milligrams; SD: Standard Deviation; TSH: Thyroid-Stimulating Hormone

Variables	Before matching								After matching							
	Cohort A	Cohort B	Patients (n) (Cohort A)	Patients (n) (Cohort B)	% of Cohort (Cohort A)	% of Cohort (Cohort B)	P-value	Standard difference	Cohort A	Cohort B	Patients (n) (Cohort A)	Patients (n) (Cohort B)	% of Cohort (Cohort A)	% of Cohort (Cohort B)	P-value	Standard difference
Age at index (years, mean +/- SD)	62.7 ± 12.4	46.1 ± 17.4	63,652	202,143	100%	100%	<0.001	1.104	60.3 ± 12.4	61.7±13.6	43,025	43,025	100%	100%	<0.001	0.106
White	-	-	37,554	98,652	59.0%	48.8%	<0.001	0.206	-	-	24,800	25,155	57.6%	58.5%	0.014	0.017
Female	-	-	45,988	159,664	72.2%	79.0%	<0.001	0.157	-	-	31,791	31,830	73.9%	74.0%	0.762	0.002
Black or African American	-	-	11,969	27,801	18.8%	13.8%	<0.01	0.137	-	-	7,361	7,130	17.1%	16.6%	0.035	0.014
Male	-	-	16,018	37,278	25.2%	18.4%	<0.001	0.163	-	-	10,079	9,945	23.4%	23.1%	0.280	0.007
Tobacco use	-	-	3,482	3,042	5.5%	1.5%	<0.001	0.217	-	-	1,792	1,693	4.2%	3.9%	0.087	0.012
Cerebral infarction	-	-	3,868	1,535	6.1%	0.8%	<0.001	0.296	-	-	1,677	1,302	3.9%	3.0%	<0.001	0.048
Ischemic heart diseases	-	-	14,858	6,760	23.3%	3.3%	<0.001	0.615	-	-	6,614	5,756	15.4%	13.4%	<0.001	0.057
Hyperlipidemia, unspecified	-	-	31,855	14,176	50.0%	7.0%	<0.001	1.084	-	-	14,369	13,189	33.4%	30.7%	<0.001	0.059
Chronic kidney disease	-	-	7,943	4,094	12.5%	2.0%	<0.001	0.412	-	-	3,631	3,215	8.4%	7.5%	<0.001	0.036
Thyroidectomy	-	-	59	592	0.1%	0.3%	<0.001	0.046	-	-	51	36	0.1%	0.1%	0.108	0.011
Radioactive iodine therapy	-	-	31	410	0.0%	0.2%	<0.001	0.043	-	-	29	29	0.1%	0.1%	1	<0.001
Propylthiouracil	-	-	1,947	3,782	3.1%	1.9%	<0.001	0.077	-	-	1,115	1,037	2.6%	2.4%	0.089	0.012
Methimazole	-	-	18,252	30,940	28.7%	15.3%	<0.001	0.327	-	-	10,673	10,474	24.8%	24.3%	0.115	0.011
Methylprednisolone	-	-	13,178	14,010	20.7%	6.9%	<0.001	0.407	-	-	7,141	7,032	16.6%	16.3%	0.316	0.007
LDL-cholesterol (mean +/- SD, mg/dL)	111.5 ± 46.4	97.9 ± 34.2	39,910	40,845	62.7%	20.2%	<0.001	0.333	117.5 ± 46.8	101.1 ± 35.5	22,186	22,122	51.6%	51.4%	<0.001	0.393
TSH (mean +/- SD, mIU/L)	5.2 ± 32.8	4.3 ± 32.1	43,603	63,407	68.5%	31.4%	<0.001	0.029	5.7 ± 35.5	4.7 ± 36.1	22,558	25,217	59.4%	58.6%	0.001	0.030
Body mass index Percentile (mean +/- SD)	30.6 ±8.1	40.8 ± 25.4	1,497	1,793	2.4%	0.9%	<0.001	0.538	30.7 ± 9.0	30.4 ± 11.7	771	666	1.8%	1.5%	0.647	0.024
HbA1c (mean +/- SD, %)	6.4 ± 1.7	5.9 ± 1.5	32,391	30,353	50.9%	15.0%	<0.001	0.350	6.5 ±1.7	6.0 ± 1.3	17,392	17,286	40.4%	40.2%	<0.001	0.334
TSI (mean +/- SD, IU/L)	128 ± 335.5	121.8 ± 250.5	4,165	9,020	6.5%	4.5%	0.234	0.021	122.1 ± 327.9	113.4 ± 237.5	2,547	2,532	5.9%	5.9%	0.828	0.001

Primary outcome

**Figure 2:**
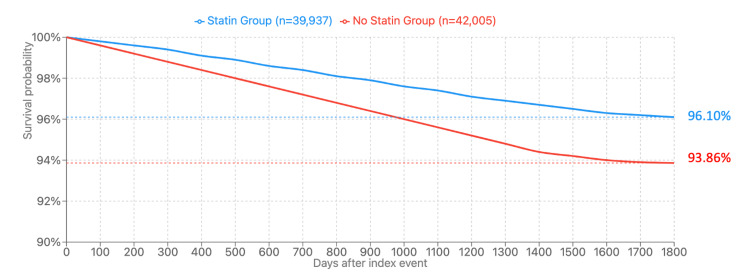
Kaplan-Meier survival curve Blue: Cohort A (treated with statins); Red: Cohort B (not treated with statins) Graphical representation of the probability of not developing thyroid eye disease (survival without the condition) between Cohort A and Cohort B. Patients with the outcome before the time window are excluded (Cohort A: n=3,088; Cohort B: n=1,020), with a total of n=39,937 in Cohort A and n=42,005 in Cohort B.  In Cohort A, n=1,171 patients developed exophthalmos, compared to n=2,047 in Cohort B.  By the end of the follow-up period (five years), 96.1% of patients in Cohort A survive without thyroid eye disease compared to 93.86% in Cohort B (hazard ratio = 0.559, 95% CI 0.521-0.601, p < 0.001).

Concerning the primary outcome of exophthalmos, it was significantly less frequent in Cohort A (exposed to statin therapy), with a notable 44% reduction in hazard (HR: 0.559, 95% CI 0.521-0.601, p < 0.001); the difference furthermore persisted over five years of follow-up. A Kaplan-Meier curve was furthermore incorporated (Figure 2) to support this finding, demonstrating a disease-free survival in Cohort A (96.1%) compared to Cohort B (93.9%). Our findings suggest a clinically meaningful impact against the progression of thyroid eye disease (specifically exophthalmos) in patients offered statins.

Secondary outcomes

Apart from exophthalmos, further disease-related complications were significantly reduced with statin exposure (Table 2). Cohort A demonstrated a decreased hazard for diplopia (HR 0.750, 95% CI 0.688-0.818, p < 0.001), suggesting statin medications may alleviate muscular imbalances and orbital fibrosis that are associated with diplopia in thyroid eye disease. Additionally, statins were shown to reduce the requirement for optic nerve surgery (HR 0.683, 95% CI 0.471-0.990, p = 0.018); although the absolute event count of this was low, these findings furthermore suggest the role of anti-inflammation and anti-fibrosis of statins. Another significant finding was the reduced requirement for teprotumumab prescriptions in Cohort A (HR 0.692, 95% CI 0.552-0.867, p = 0.047), which just reached statistical significance; teprotumumab is typically reserved for moderate-to-severe thyroid eye disease, and therefore, the reduced prescription may suggest statins lead to milder disease progression.

**Table 2:** Primary and secondary outcomes X^2^: Chi-Square Primary outcome: Development of thyroid eye disease (exophthalmos) demonstrated a statistically significant reduced hazard over five years. Secondary outcomes are notable for a significant reduction in optic nerve surgery, diplopia, and teprotumumab usage. No significant differences were noted for color vision impairment, blindness, optic nerve injury, strabismus or ophthalmology referrals.

Primary outcome	Number of patients excluded and outcome	Hazard ratio	95% confidence interval	P-value	X^2^
Exophthalmos	Cohort A: 3,088 excluded (Outcome: 1,171 out of 39,937); Cohort B: 1,020 excluded (Outcome: 2,047 out of 42,005)	0.559	0.521-0.601	0.000	5.644
Secondary outcomes	Number of patients excluded and outcome	Hazard ratio	95% confidence interval	P-value	X^2^
Optic nerve surgery	Cohort A: 37 excluded (48 out of 42,988); Cohort B: 10 excluded (67 out of 43,017)	0.683	0.471-0.990	0.018	5.644
Optic nerve injury	Cohort A: 382 excluded (216 out of 42,643); Cohort B: 167 excluded (234 out of 42,858)	0.859	0.713-1.033	0.011	6.531
Color vision impairment	Cohort A: 15 excluded (16 out of 43,010); Cohort B: 17 excluded (18 out of 43,008)	0.806	0.411-1.581	0.260	1.271
Diplopia	Cohort A: 1,717 excluded (918 out of 41,308); Cohort B: 953 excluded (1,154 out of 42,072)	0.750	0.688-0.818	0.000	14.490
Blindness and low vision	Cohort A: 935 excluded (705 out of 42,090); Cohort B: 576 excluded (576 out of 42,306)	1.114	0.998-1.244	0.002	9.362
Strabismus	Cohort A: 13 excluded (10 out of 43,012); Cohort B: 10 excluded (11 out of 43,023)	0.331	0.105-1.041	0.819	3.956
Teprotumumab	Cohort A: 74 excluded (133 out of 42,951); Cohort B: 17excluded (176 out of 43,008)	0.692	0.553-0.867	0.047	10.343
Ophthalmology referral	Cohort A: 85 excluded (61 out of 42,940); Cohort B: 46 excluded (34 out of 42,979)	T-test: 0.734	Mean (SD): Cohort A: 1.607 (1.215); Cohort B: 1.441 (0.660)	0.465	N/A

Other outcomes, such as color vision impairment (HR 0.806, 95% CI 0.411-1.581, p = 0.260), optic nerve injury (HR 0.859, 95% CI 0.713-1.033, p = 0.011), strabismus (HR 0.331, 95% CI 0.105-1.041, p = 0.819), and blindness (HR 1.114, 95% CI 0.998-1.244, p = 0.002), failed to reach statistical significance. This suggests either a limited role in the usage of statins for preventing these endpoints, or that a longer up time beyond five years (along with a larger sample size to detect subtle effects) is needed, as these complications may develop slowly, and may not have been captured in this time frame.

Interestingly, referrals to ophthalmology (both self and provider-directed) were not statistically significant between cohorts (Cohort A mean referral 1.607, Cohort B mean referral 1.441, t-test statistic = 0.734, p = 0.465), suggesting statin use did not influence the likelihood for specialist evaluation. Of note, however, this may be due to system-level referrals rather than disease severity alone.

## Discussion

The findings of our study are consistent with available literature, noting a beneficial effect of statins with respect to the development of thyroid eye disease and associated outcomes. Whereas other studies are limited in geographic location, this study is the first to provide a global estimate (subject to TriNetX availability), allowing for a larger sample size. Our literature search analyzed seven further studies (one randomized controlled trial and six observational studies), all demonstrating a consistent, favorable effect of statin medications (Table 3). Stein et al. [7] suggest that at least 60 days of statin usage in the preceding year leads to a near 40% reduction in the development of thyroid eye disease in an American observational study (HR 0.6, 95% CI 0.37-0.93). The authors furthermore note non-statin based anti-lipidemic agents, and other anti-inflammatory agents (such as cyclo-oxygenase inhibitors) fail to demonstrate such effects, suggesting the effect is class (statin)-specific. The latter finding is concurred by Nilsson et al. [8] in a Swedish study, noting a class-specific effect of statins (Atorvastatin and Simvastatin) (HR 0.74, 95% CI 0.65-0.84), irrespective of type or dose. In a study performed in Taiwan by Chou et al. [9], a class-effect of statins is again found to reduce the risk of thyroid eye disease, irrespective of the type of statin or intensity (HR 0.79, 95% CI 0.63-0.99). Lee et al. [10] perform an observational study in Korea, suggesting solely higher-statin dosages are associated with a reduced risk of thyroid eye disease in women (but not men, HR 0.37, 95% CI 0.22-0.62), however, this finding has not been replicated in further studies; in fact, Nilsson et al. [8] noted a stronger correlation with men in their study. In another study performed in Taiwan, Hsu et al. [11] again, note a protective effect with statins (OR 0.19, 95% CI 0.10-0.39). Reynolds et al. [12] differ from the observational studies, whereby the role of statin therapy in pre-established thyroid eye disease (with strabismus) is assessed in this American study; via retrospective analyses, the authors note that patients treated with statins are less likely to require decompression surgery. Lazolla et al. [13] have to date performed the only randomized controlled trial (in Italy), assessing the outcomes of thyroid eye disease in pre-existent patients, stratified into treatment with intravenous glucocorticoids and atorvastatin versus monotherapy with intravenous glucocorticoids, noting a significantly reduced attributable risk in composite outcomes of exophthalmos, clinical activity score, eyelid aperture, and diplopia (AR 0.23, 95% CI 0.02-0.44).

**Table 3:** Characteristics of analyzed studies Review of the available literature about the effects of statins and thyroid eye disease. Our search demonstrated seven studies that have directly assessed the effect of statins and thyroid eye disease. Currently, there are five observational studies assessing statins for prevention of thyroid eye disease, one observational study assessing the impact of statins with pre-established strabismus, noting rates of decompression/strabismus surgery, and one randomzied controlled trial assessing statins and intravenous glucocorticoids (versus monotherapy with intravenous corticosteroids) for a multitude of clinical outcomes concerning thyroid eye disease.

Authors	Year	Country	Population	Follow-up	Intervention	Cases and controls	Study design	Outcome	Findings
Stein et al. [7]	2015	USA	Newly diagnosed Graves’ disease, age > 18	5.6 years	Statin medication (type not specified)	Cases: 740 Controls: 7,664	Retrospective cohort	Thyroid eye disease development	HR 0.60 (95% CI 0.37-0.93) with statin usage for > 60 days over past year.
Nilsson et al. [8]	2020	Sweden	Newly diagnosed Graves’ disease, age > 18	4.5 years	Simvastatin or Atorvastatin	Cases: 5,574 Controls: 34,409	Retrospective cohort	Thyroid eye disease development	HR 0.74 (0.65-0.84) for development of thyroid eye disease.
Chou et al. [9]	2025	Taiwan	Graves’ disease, age > 40	4.4 years	All statins	Cases: 7,073 Controls: 95,785	Retrospective cohort	Thyroid eye disease development	HR 0.79 (95% CI 0.63-0.99) for developing Graves’ Ophthalmopathy
Lee et al. [10]	2023	Korea	Newly diagnosed Graves’ disease	102 months for women	All statins (not specified)	Cases (Female): 293, Control (Female): 5,047	Retrospective cohort	Thyroid eye disease development	In women, higher statin dose associated with reduced risk of thyroid eye disease (HR 0.37, 95% CI 0.22-0.62).
Hsu et al. [11]	2024	Taiwan	Newly diagnosed Graves’ disease, age > 20	62.6 months	Statin (type not specified)	Cases: 372 Controls: 3,206	Retrospective cohort	Thyroid eye disease development	OR 0.19 (95% CI 0.10-0.39) for development of thyroid eye disease amongst statin users.
Reynolds et al. [12]	2018	USA	Thyroid eye disease and Strabismus	20 months	Statin (x4 Simvastatin, x3 Atorvastatin, x3 Pravastatin, x1 Lovastatin, x1 unknown statin)	Cases: 12 Controls: 18	Retrospective cohort	Decompression surgery or Strabismus surgery	Significantly lower decompression surgeries with statin users (p = 0.04).
Lanzolla et al. [13]	2021	Italy	Active thyroid eye disease	24 weeks	Atorvastatin + Intravenous Glucocorticoid	Cases: 41 Controls: 39	Randomized controlled trial	Exophthalmos, eyelid aperture, diplopia, clinical activity score (composite outcome)	Attributable Risk 0.23 (95% CI 0.02-0.44) for composite outcomes.
Our study	2025	Global	Newly diagnosed Graves’ disease, age > 18	5 years	All statins (not specified)	Cases: 43,025 Controls: 43,025	Retrospective cohort	Thyroid eye disease development	HR 0.559 (95% CI 0.521-0.601) for the development of thyroid eye disease.

Statins are a potent class of medications, acting to inhibit 3-Hydroxy-3-methylglutaryl-coenzyme A reductase (HMG-CoA Reductase), preventing the development of low-density-lipoprotein (LDL) cholesterol, and subsequent cardiovascular risk reduction. Within the literature, there is inconsistent evidence of a significant correlation between elevated cholesterol (both total and LDL) and thyroid eye disease. Due to the findings from prior studies suggesting the reduction in thyroid eye disease appears to be statin-specific, it is hypothesized that the mechanism of reduced orbitopathy is independent of the lipid-lowering properties of statins. The exact underlying mechanism supporting the role of statins remains poorly understood. In addition to lipid-lowering properties, statins are known to exhibit anti-inflammatory properties, as demonstrated in the JUPITER study (with reduction in circulating high-sensitivity C-reactive protein) [14]. Furthermore, statins are known to induce apoptosis (via prevention of mevalonate production). Koch et al. [15] suggest that statins may convert inflammatory T-cells into an anti-inflammatory response via up-regulation of Th2 cells. Nilsson et al. [8] suggest statins may mobilize pro-inflammatory T-cells into the blood (from the diseased site), leading to remission. The authors furthermore suggest that statins function to reduce adipogenesis (associated with systemic inflammation). Statins are also known to inhibit myofibroblast differentiation in orbital fibroblasts in the setting of transforming growth factor-beta, which may explain a direct anti-fibrotic role [11]. Insulin-like growth factor 1 (IGF-1) is known to play a role in the development of thyroid eye disease, with IGF-1 receptors the site of action of teprotumumab; Chou et al. [9] note atorvastatin lowers levels of Insulin-like growth factor 1 (IGF-1), which is involved in thyroid eye disease, and note simvastatin and pravastatin inhibit IGF-1 receptor signaling. As a final note, Lanzolla et al. [13] suggest that a synergistic anti-inflammatory effect is evident between statins and anti-inflammatories (such as glucocorticoids).

Limitations in our study exist, most notably its dependence upon the TriNetX software for analysis. TriNetX relies on adequate clinician documentation and coding, which can often be a source of error in electronic medical records. Another limitation is the fact that we did not set a minimum number of days treated with a statin, and therefore a range of (both compliant and non-compliant) patients are included in Cohort A. Similarly, the dosages and individual statin medications were not separated and analyzed individually; additionally, past users of statin medications were excluded in both groups. While we did not assess other anti-lipidemic agents, this has been analyzed by other authors (as noted above). The mean LDL and HbA1c in cohort A remained significantly higher compared to cohort B, for which it would be expected to have an increased prescription of statin medications due to higher cardiovascular risk, however, despite the elevated HbA1c (which itself is a risk factor for thyroid eye disease) which would expect to bias the results towards the null, the primary outcome remained significantly lower. Due to the nature of this study design (retrospective and observational), unmeasured confounders are likely to have influenced our results and cannot be excluded. As with other studies, patients who received statin medications are likely to have differed systematically from those who did not (even after adjustment with propensity score matching because of residual confounders). While we analyzed up to five years, it is unknown whether the protective effect of statins continues beyond this time frame (similar to “metabolic memory” with intensive glucose control at diagnosis of diabetes mellitus), for which further studies are required to investigate this phenomenon. Similarly, while outcomes, such as strabismus, optic nerve injury, and color vision, were not significant, this may reflect the duration of our study (five years), for which further studies are required to assess such outcomes over a longer period (with an increase in sample size to detect subtle differences). With our primary outcome of exophthalmos, we were unable to stratify the “severity” and “activity”, a common limitation reported by other studies. Our findings are based on a pre-defined population on the TrinNetX Network, which may not be representative of many thyroid eye disease patients globally. Regarding the secondary outcome of teprotumumab, we were unable to adjust for geographic location (teprotumumab is available only in the United States of America) and insurance status (teprotumumab is an expensive medication, which requires coverage by insurance), both of which are likely to confound our findings. While this study involves global datasets, the majority of patients within each cohort were located within the United States of America, limiting its widespread interpretation and applicability to other countries.

## Conclusions

Our propensity score-matched, retrospective cohort study demonstrated a statistically significant protective association regarding statins and the development of thyroid eye disease in patients with pre-existing Graves' disease. At five years, statin usage was associated with a near 44% reduction in the hazard of exophthalmos, as well as decreased rates of teprotumumab usage, optic nerve surgery, and diplopia. Such benefits appear to be statin specific as they have not been replicated with other (non-statin) anti-lipid agents, suggesting the benefit is beyond their effect upon cholesterol. Further randomized controlled trials are required to define the optimal dosing regimen, provide data on long-term efficacy, elucidate the underlying mechanisms, and confirm causality.
